# Reduced left dorsolateral prefrontal activation and right inferior frontal de-oxygenation differ between psychotic and non−psychotic adolescent depression during verbal fluency

**DOI:** 10.3389/fpsyt.2026.1689631

**Published:** 2026-06-02

**Authors:** Gang Liu, Yu Yang, Jie Liao, Yanping Feng, Sha Zhang, Yudiao Liang

**Affiliations:** Department of Psychiatry, Zigong Hospital Affiliated to Southwest Medical University, Zigong, China

**Keywords:** fNIRS, major depressive disorder, prefrontal cortex, prefrontal hemodynamics, psychotic symptoms, verbal fluency

## Abstract

**Background:**

Adolescents with major depressive disorder (MDD) who exhibit psychotic symptoms (MDD-Psy) often show greater clinical severity and higher suicide risk than those without psychosis (MDD-NonPsy). It remains unclear whether prefrontal hemodynamic responses during cognitive challenge can differ between these subgroups.

**Methods:**

We recruited 273 adolescents aged 14–16 years (110 MDD-NonPsy, 163 MDD-Psy). All completed a 60-s verbal fluency task (VFT) while 53-channel functional near-infrared spectroscopy recorded oxy- and deoxyhemoglobin (HbO, HbR) from six frontal ROIs. Whole-brain FDR correction identified baseline-deviated channels, and ROC analyses tested the discriminative value of significant channels/ROIs. Clinical severity was indexed by the Kutcher Adolescent Depression Scale (KDAS) and the Brief Psychiatric Rating Scale (BPRS).

**Results:**

Compared with MDD-NonPsy, adolescents with MDD-Psy exhibited higher KDAS (median 16 vs 13; z = -7.07, p < 0.001) and BPRS total scores (median 35 vs 28; z = -11.02, p < 0.001). After FDR correction, MDD-NonPsy showed positive deviations at channels 8 and 23 (t = 2.10-2.70, pFDR=0.038), whereas MDD-Psy exhibited negative deviation at channel 2 (MNI –60, 19, 18; left middle frontal gyrus; t =-2.89, pFDR = 0.004). Across ROIs, only right inferior frontal gyrus HbR differed between groups (median –0.03 vs 0.00 mM·mm; z = -2.18, pFDR = 0.030). No ROI-level HbO differences survived correction. At the single-channel level, HbO at channel 2 and HbR in IFG_R correlated modestly with lower KDAS and restlessness scores (ρ = –0.14 to –0.16, p <0.050). ROC analyses indicated modest but significant classification accuracy for psychosis (AUC = 0.577-0.581, p = 0.030).

**Conclusions:**

Left dorsolateral and right inferior prefrontal hemodynamic signatures differ between adolescents with MDD who present with versus without psychotic features. However, given the absence of healthy control or other psychiatric comparison groups, these findings cannot establish diagnostic specificity. Pending replication in independent samples, these fNIRS−derived hemodynamic signatures may inform future hypothesis−driven research.

## 1 Introduction

Depressive disorders impose a staggering global health burden, with recent epidemiological studies estimating that approximately 280 million individuals worldwide suffer from major depressive disorder (MDD) (1, 2). This condition accounts for 4.7% of all disability-adjusted life years (DALYs), positioning it among the leading causes of functional impairment globally (3). Within this heterogeneous diagnostic category, psychotic depression (MDD-Psy) emerges as a particularly pernicious subtype, characterized by co-occurring delusions or hallucinations alongside depressive symptoms (4). Epidemiological studies report that 9.2–30% of individuals with MDD present psychotic features, and this proportion increases with illness severity manifested by frequent relapses, suicidality, and treatment resistance (4, 5). Critically, MDD-Psy demonstrates distinct clinical trajectories, including elevated suicide risk, greater functional impairment, and poorer response to conventional therapies compared to non-psychotic MDD (MDD-NonPsy) (4–7). In adolescents, this subtype is further associated with heightened severity scores on depression and psychosis rating scales [e.g., the Kutcher Adolescent Depression Scale (KDAS) and the Brief Psychiatric Rating Scale (BPRS)], underscoring the urgency for early neurobiological differentiation (8). Clinical cohort studies further demonstrate that psychotic features are associated with a two- to three-fold increase in relapse probability and a disproportionately high burden of cognitive and psychomotor impairment (9). Despite these well-characterized clinical risks, current diagnostic frameworks remain purely symptom-based (DSM-5 or ICD-10), and no validated biomarkers exist to objectively identify MDD-Psy or to guide targeted intervention (4, 10).

The dorsolateral prefrontal cortex(DLPFC) and inferior frontal gyrus (IFG) are key nodes for cognitive control, working-memory maintenance, and top-down regulation of affect (11, 12). Meta-analyses of randomized trials show that high-frequency repetitive transcranial magnetic stimulation (rTMS) over the left DLPFC yields a medium-to-large effect on depressive symptoms (Hedges’ g=-0.73), whereas right-DLPFC continuous theta-burst stimulation (cTBS) preferentially reduces suicidal ideation (13, 14). These clinical data are paralleled by mechanistic findings that stress and inflammation disrupt DLPFC layer-III microcircuits through kynurenic-acid-mediated NMDA-receptor blockade and aberrant calcium-cAMP signaling, leading to impaired cognitive flexibility (12). Despite convergent evidence for DLPFC/IFG dysfunction in MDD, the extent to which these alterations differ between psychotic and non-psychotic subtypes remains unresolved. Resting-state fMRI studies reveal that patients with psychotic features exhibit exaggerated DLPFC–default-mode coupling and weakened DLPFC–limbic connectivity (15), patterns that align with the negative hemodynamic deviation we observed in the left DLPFC during verbal fluency. Moreover, the right IFG de-oxyhemoglobin reduction detected in MDD-Psy correlates with heightened psychomotor activation, suggesting a lateralized deficit in inhibitory control. Collectively, these observations indicate that DLPFC and IFG dysfunction may not only underpin core depressive pathology but also encode subtype-specific neurophysiological signatures, offering a rationale for fNIRS-guided stratification and targeted neuromodulation in high-risk youth.

Portable, motion-robust functional near-infrared spectroscopy (fNIRS) has rapidly become a preferred tool for probing prefrontal cortex (PFC) function in youth psychiatric research (16, 17). Its silent, wearable design minimizes the scanning anxiety and motion artifacts that frequently compromise EEG or fMRI in adolescents (18, 19). The 60-s verbal fluency task (VFT) is now the canonical paradigm for evoking well-characterized oxyhemoglobin and deoxygenated hemoglobin (HbO, HbR) responses across DLPFC and inferior frontal gyrus (IFG) regions (20–22), and has been validated in both clinical (21, 23) and sub-clinical adolescent samples (2). However, a decisive research gap persists: To our knowledge, no fNIRS investigation has yet determined whether VFT-evoked hemodynamic signatures differentiate adolescents with MDD-Psy from those with MDD-NonPsy. Current evidence demonstrates global VFT hypofrontality in adolescent depression (20, 22), abnormal IFG activation in self-harm subgroups (23), and age-dependent DLPFC modulation (22), yet the unique neurovascular profile of MDD-Psy remains uncharacterized. Filling this lacuna is essential to transform fNIRS from a research curiosity into a scalable, bedside biomarker for the early detection of the most severe MDD subtype and for guiding personalized neuromodulation.

The overarching goal of the present study is to determine whether fNIRS-derived hemodynamic responses during a 60-s VFT can differ between adolescents with MDD-Psy from those with MDD-NonPsy. We hypothesized that, relative to MDD-NonPsy, the MDD-Psy group would show significantly reduced oxyhemoglobin activation (negative ΔHbO) in the left dorsolateral prefrontal cortex and an atypical, more negative deoxyhemoglobin shift in the right inferior frontal gyrus. Further, we expected the magnitude of these aberrant hemodynamic signals to correlate positively with clinical severity (KDAS total, BPRS thought-disturbance and restlessness sub-scores).

## 2 Methods

### 2.1 Patients and experimental protocol

We consecutively recruited inpatients who met DSM-5 criteria for a current major depressive episode at Zigong Mental Health Center between May 2024 and February 2025. Inclusion criteria: meeting the Diagnostic and Statistical Manual of Mental Disorders, 5th edition (DSM-5) criteria for current major depressive episode, confirmed by Mini International Neuropsychiatric Interview (Mini), KDAS total score≥12, right-handed (confirmed by the Edinburgh Handedness Inventory-Short Form, EHI-SF, with a laterality quotient > +40), and able to complete the experimental tasks. Family socioeconomic status (SES) was estimated based on the highest educational level attained by either parent (categorized as: junior high school or below, senior high school, and college or above). No patient was excluded solely on the basis of current or prior psychotropic medication use; both medicated and unmedicated individuals were eligible. MDD-Psy was defined as the presence of hallucinations or delusions on the MINI Psychosis module. Exclusion criteria comprised any neurologic disorder, full-scale IQ < 70 (assessed using the Chinese version of the Wechsler Abbreviated Scale of Intelligence, Second Edition, WASI-II), or prior repetitive transcranial magnetic stimulation. Patients with a history of other neuromodulation interventions (e.g., electroconvulsive therapy [ECT], transcranial direct current stimulation [tDCS], vagus nerve stimulation [VNS]) were also excluded. The procedures followed in this experiment were approved by the Ethics Committee of Zigong Hospital Affiliated to Southwest Medical University. After signing the informed consent form, participants completed the Chinese version of the Kutcher Adolescent Depression Scale-11 (24) (KANDS-11) and the Brief Psychiatric Rating Scale-18 (25) (BPRS) for clinical characteristics assessment. KADS−11 has demonstrated excellent internal consistency (Cronbach’s α = 0.89) and test–retest reliability (ICC = 0.85) in Chinese adolescents. The BPRS has been widely used in Chinese psychiatric populations and has shown good inter−rater reliability (ICC = 0.81-0.93 across subscales) and concurrent validity with the Positive and Negative Syndrome Scale (PANSS). These validation studies support the suitability of both instruments for the present study.

All clinical assessments were conducted by two trained psychiatrists (J.L. and S.Z.) with at least three years of experience in adolescent psychiatry. Prior to study initiation, both raters completed a structured training program consisting of: (i) didactic sessions on DSM−5 diagnostic criteria for major depressive disorder and the psychotic depression specifier; (ii) joint rating of 20 videotaped diagnostic interviews with consensus scoring; and (iii) supervised administration of the KADS−11 and BPRS−18 in 10 pilot patients not included in the final sample.

During the main study, inter−rater reliability was assessed by having both raters independently evaluate a randomly selected subset of 40 participants (14.6% of the total sample). Ratings were conducted on the same day, and raters were blinded to each other’s scores. Intraclass correlation coefficients (ICC; two−way random effects model, absolute agreement) were computed for KADS−11 total scores and BPRS total and subscale scores. The inter−rater reliability was excellent for KADS−11 total scores (ICC = 0.94, 95% CI: 0.89–0.97) and for BPRS−18 total scores (ICC = 0.91, 95% CI: 0.84–0.95). Subscale ICCs ranged from 0.82 (Activation) to 0.93 (Thought Disturbance), indicating consistently high agreement. These results confirm the reliability of the clinical groupings used in this study.

During the experiment, participants sat comfortably in a quiet room and underwent VFT (26, 27) while their cortical activation was measured using fNIRS. The VFT paradigm followed a block design consisting of three phases: (i) a 30 s pre task resting baseline, during which participants were instructed to relax and count silently; (ii) a 60 s task period, during which participants generated words aloud; and (iii) a 60 s post task resting baseline, during which participants again rested and counted silently. Throughout the task, participants were instructed to use four Chinese characters to generate as many Chinese phrases as possible, such as “tian” (“ day “), “bai” (“ white “), “shu” (“ tree “), and “hua” (“ flower “). During this period, automatic changes were made every 15 s. All patients received detailed instructions about the experimental procedures before the study.

Prior to the fNIRS recording, all participants completed a brief practice block consisting of two 15-s trials using the stimulus character (kǒu, “mouth”). During this practice, the experimenter verified that the participant understood the instruction to generate as many Chinese phrases as possible beginning with the given character. Participants who failed to generate at least two phrases during the practice block were given additional explanation and a second practice trial. No participant was excluded due to inability to perform the task after this brief training. Throughout the task, an experimenter remained in the room to monitor compliance and provide non-directive encouragement (e.g., “keep going”) if the participant paused for >10 s.

Medication status at the time of assessment was systematically recorded for all participants. Current use of antidepressants, antipsychotics, and mood stabilizers was documented based on clinical records and confirmed by patient/parent interview. Patients were classified as currently medicated (receiving a stable dose for ≥2 weeks) or unmedicated (no psychotropic medication in the preceding 4 weeks). Medication status was included as a covariate in subsequent sensitivity analyses.

### 2.2 NIRS measurement

Cortical activation was measured using a 53-channel fNIRS device (BS-3000, Wuhan Znion Medical Technology Co., LTD., Wuhan, China) with a sampling rate of 20 Hz. The near-infrared wavelengths used were 690 and 830 nm, and the optoelectronic devices were arranged as 16 sources/16 detectors. Channels were placed according to the 10–20 system, with source-detector pairs separated by 3 cm (source 9 within FPz). Before each measurement, the fNIRS system was calibrated, and testing was started only when the repetition rate exceeded 90%. Channel distribution is shown in **Supplementary Material 1**. A 3-D digitizer (Wuhan Znion Medical Technology Co., Wuhan, China) recorded Montreal Neurological Institute (MNI) coordinates (28); channels were later mapped to six anatomical regions of interest (ROIs) using the NIRS-SPM toolbox (29).

### 2.3 Data analysis

#### 2.3.1 Data pre-processing

First, the data sampling rate was reduced to 10Hz. The Coefficient of Variation (CV) value of the data was calculated with 10s before the task as the baseline. If the CV value of a certain channel is greater than 25%, the channel is considered a bad channel. CV values were calculated for each task cycle, and a channel was considered as a bad channel if its CV was greater than 25% (30). With labeling as the starting point, overlay averaging was performed 2 s before and 60 s after each labeling, and this block of data was baseline calibrated 2 s before labeling. First, using the Matlab-based toolkit Homer2, using the modified Beer-Lambert law in Homer2 (31), Raw optical density was converted to HbO\HbR concentration changes to convert raw light intensity data to optical density. tMotion was assigned 0.3 seconds, tMask was assigned 3 seconds, and the remaining motion correction parameters were left as default. To detect and fix motion artifacts, the procedure involves applying a moving standard deviation combined with cubic spline interpolation. A bandpass filter between 0.01 and 0.1 Hz was used to eliminate physiological noise, including low-frequency drift and high-frequency noise. Changes in the concentrations of HbO and HbR were calculated according to the modified Beer-Lambert law (32). Participants with > 30% bad channels were excluded (n = 2), leaving 273 datasets for analysis.

#### 2.3.2 General linear model (GLM) analysis

We examined task-evoked activation during VFT using the GLM (33, 34) method and the Matlab-based toolbox NIRS-KIT (35). The experimental design matrix includes a constant term and task regressors constructed by convolving a van function with the classical hemodynamic response function (HRF) (36). We derived task β values reflecting task-evoked activation and used them in group-level statistical analyses (29). We selected Oxy-Hb and deoxy-Hb as the primary measures in our subsequent analyses. Oxy-Hb was explicitly included as the primary metric due to its higher signal-to-noise ratio (SNR) and better sensitivity in detecting cortical activation (37, 38). Although the deoxy-Hb signal was weak, its change pattern was highly consistent with the BOLD signal of fMRI (39). Given that previous studies reported a lack of satisfactory stability of the signal at the single-channel level, we performed an analysis at the ROI (region of interest) level (40–42). For each channel, the integral value over a 60-second task period was calculated and averaged within a given ROI to be used as ROI metrics. The threshold for group-level activation maps was p < 0.05 (FDR corrected).

#### 2.3.3 ROI-level feature extraction

The changes of Oxy-Hb and deoxyhemoglobin concentrations in each channel during the 60-s task were calculated. The average of all channels within a given ROI was calculated as the ROI metric, and any channel with a CV value of more than 25% was considered a “bad channel” and would be omitted from the ROI calculation. Six rois were identified based on the automated anatomical labeling (AAL) probability map: left inferior frontal gyrus (IFG_L), left middle frontal gyrus (MFG_L), left superior frontal gyrus (SFG_L), right superior frontal gyrus (SFG_R), right middle frontal gyrus (MFG_R), and right inferior frontal gyrus (IFG_R). Concentration changes in these ROIs were analyzed to assess task-related activation. The distribution of ROI is shown in **Supplementary Material 2**. Spatial registration of fNIRS channels to cortical structures was performed using the NIRS−SPM toolbox implemented in MATLAB. First, the 3D digitizer coordinates of each channel were recorded in the subject’s native head space and then transformed into Montreal Neurological Institute (MNI) standard space using the probabilistic registration method described by Singh et al. For each channel, the toolbox computed the probability of belonging to each AAL region based on a digital probability map. A channel was assigned to a given ROI if the posterior probability exceeded 50%.

### 2.4 Statistical analysis

All statistical analyses were conducted with IBM SPSS Statistics, version 26.0 (IBM Corp., Armonk, NY, USA). The Chi-square test or Mann-Whitney U test was used for demographic comparison. A one-sample t-test was performed on task β values to evaluate the group-level task-evoked activation in each group and within the channel. Between-group differences in activation were calculated with the use of two-sample t-tests, and statistical tests were corrected for multiple comparisons using the false discovery rate (FDR). For integrated values at the ROI level, Mann-Whitney tests were performed on each ROI. Spearman correlation analysis was used to analyze the correlation between ROI features and clinical scores (KDAS, BPRS). Receiver operating characteristic (ROC) analysis was performed to test the discriminative power (AUC) to distinguish MDD-Psy from MDD-NonPsy. All were two-tailed tests, and p < 0.05 was considered statistically significant.

## 3 Results

### 3.1 Baseline demographic and clinical characteristics

Among 273 adolescents with a major depressive episode (**Table 1**), 110 had no psychotic symptoms (MDD-NonPsy) and 163 had psychotic symptoms (MDD-Psy). The two groups did not differ in sex distribution (χ² = 2.89, P = 0.089) age (z = –2.998, P = 0.003) or illness duration (all p > 0.05). However, the MDD-Psy group had a significantly higher proportion of recurrent episodes (63.2% vs. 40.0%, χ² = 14.26, p < 0.001).KDAS total scores were higher in MDD-Psy (median 16, IQR 3) than MDD-NonPsy (13, 3; z=–7.074, P<0.001), driven by “lack of drive” (7 vs 6; z=–6.692, P<0.001) and “restlessness” (8 vs 7; z=–4.705, P<0.001). BPRS total scores were also elevated in MDD-Psy (35, 5) versus MDD-NonPsy (28, 2; z=–11.020, P<0.001), with significant increases in “thought disturbance” (8 vs 4; z=–12.450, P<0.001), “hostility” (6 vs 3; z=–8.745, P<0.001) and “activation” (3 vs 3; z=–3.939, P<0.001); “anxiety–depression” and “emotional withdrawal” did not differ (P≥0.220). The median number of words generated during the 60-s VFT was 12 (IQR: 9–14) in the MDD-NonPsy group and 11 (IQR: 9–14) in the MDD-Psy group. The difference was not statistically significant (z = –1.18, p = 0.238). This indicates comparable task engagement and verbal productivity between the two groups, and suggests that the observed hemodynamic differences are unlikely to be attributable to differential effort or task difficulty.

**Table 1:** Demographic and clinical characteristics of adolescents with major depressive episode, stratified by psychotic symptoms.

Characteristic	MDD-NonPsy (n = 110)	MDD-Psy (n = 163)	Statistic	P value
Sex, n (%)			χ² = 2.89	0.089
Male	24 (21.8%)	23 (13.9%)		
Female	86 (78.2%)	142 (86.1%)		
KDAS total score	13 (3)	16 (3)	z=–7.074	< 0.001^**^
Lack of drive	6 (1)	7 (2)	z=–6.692	< 0.001^**^
Restlessness	7 (1)	8 (2)	z=–4.705	< 0.001^**^
BPRS total score	28 (2)	35 (5)	z=–11.020	< 0.001^**^
Anxiety–depression	14 (1)	14 (1)	z=–1.227	0.220
Emotional withdrawal	4 (0)	4 (0)	z=–1.082	0.279
Thought disturbance	4 (1)	8 (2)	z=–12.450	< 0.001^**^
Activation	3 (0)	3 (1)	z=–3.939	< 0.001^**^
Hostility	3 (1)	6 (3)	z=–8.745	< 0.001^**^
Age (years)	15 (2)	15 (2)	z=–2.998	0.003**
Illness duration, months	8.5 (4.0-14.0)	9.0 (5.0-16.0)	z = -1.24	0.215
Episode status, n (%)			χ² = 14.26	<0.001
First episode	66 (60.0%)	60 (36.8%)		
Recurrent episode	44 (40.0%)	103 (63.2%)		
Medication status, n (%)				
Antidepressant use	42 (38.2%)	71 (43.6%)	0.76	0.383
Antipsychotic use	11 (10.0%)	63 (38.7%)	26.75	<0.001
Mood stabilizer use	5 (4.5%)	18 (11.0%)	3.51	0.061
Unmedicated	58 (52.7%)	46 (28.2%)	16.44	<0.001
VFT word count, median (IQR)	12 (9–14)	11 (9–14)	z = –1.18	0.238

MDD-NonPsy, major depressive episode without psychotic symptoms; MDD-Psy, major depressive episode with psychotic symptoms; KDAS, Kutcher Depression Adolescent Scale; BPRS, Brief Psychiatric Rating Scale. non-normally distributed data were presented as median (interquartile range, IQR); VFT, verbal fluency task. *p < 0.05, **p < 0.01.

### 3.2 FDR-corrected baseline-deviated VFT activation patterns in adolescent MDD with and without psychotic symptoms

**Figure 1:**
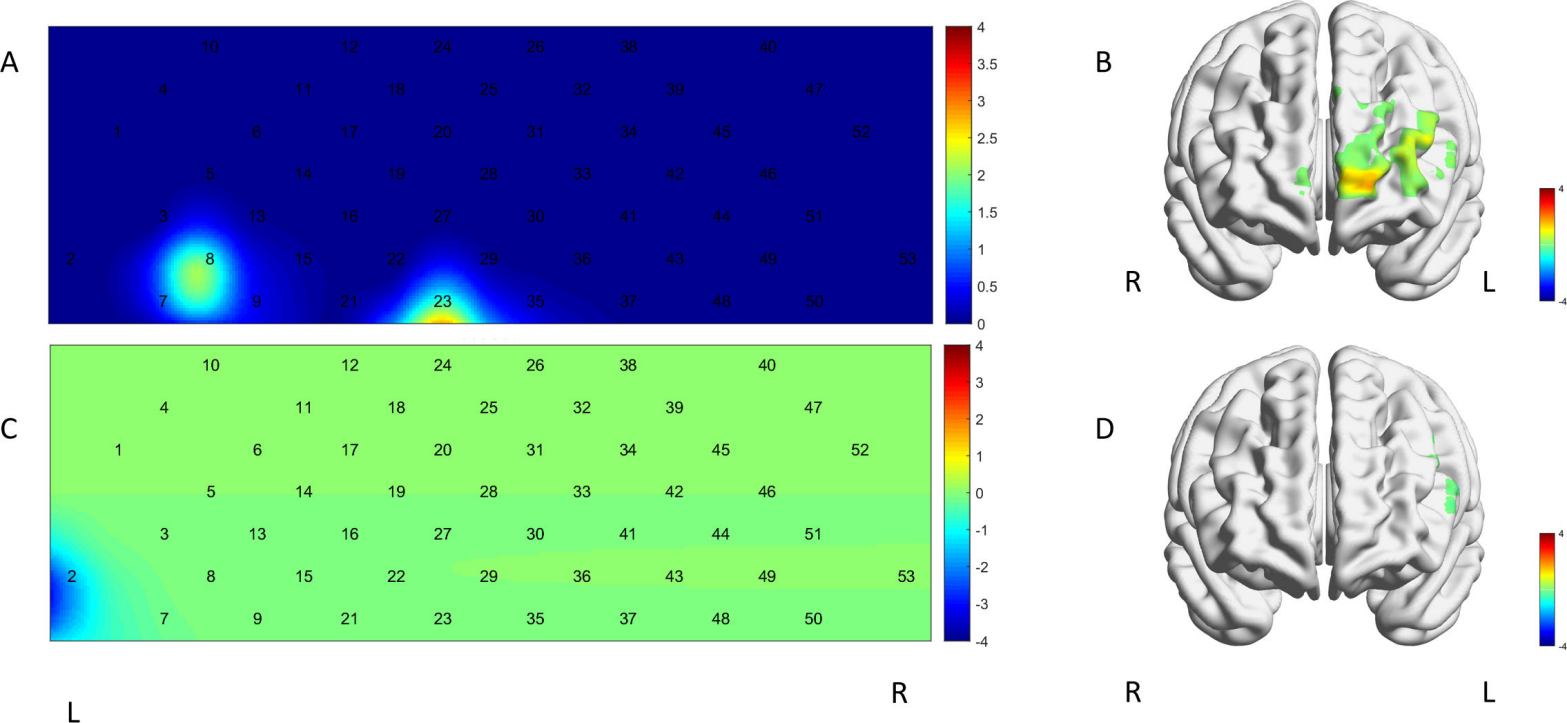
Group-level FDR-corrected activation maps during the VFT. **(A)** Two-dimensional and **(B)** three-dimensional renderings show significant activation in adolescents with MDD-NonPsy. **(C)** Two-dimensional and **(D)** three-dimensional renderings show significant negative activation in adolescents with MDD-Psy. Warm colors indicate positive deviation from baseline; cool colors indicate negative deviation from baseline. Spatial correspondence with channels 8, 23, and 2 in **Table 2** is highlighted.

After FDR correction across the whole brain, significant deviations from baseline during the VFT emerged at three channels (**Table 2**). In MDD-NonPsy, channels 8 (MNI: –52, 41, 12) and 23 (MNI: –13, 73, –2) showed significant positive deviations (channel 8: t = 2.10, pFDR = 0.038; channel 23: t = 2.70, pFDR = 0.008), whereas channel 2 (–60, 19, 18) showed a negative deviation in the MDD-Psy group (t = –2.89, pFDR = 0.004). **Figure 1** displays the activation patterns of all channels for both patient groups during the VFT.

**Table 2:** FDR-corrected significant activation differences during the VFT task.

Channel	MNI Coordinates (x, y, z)	Statistic	p-value (FDR)	Direction*
8	(-52, 41, 12)	t = 2.10	0.038	↑ (MDD-NonPsy)
23	(-13, 73, -2)	t = 2.70	0.008	↑ (MDD-NonPsy)
2	(-60, 19, 18)	t =-2.89	0.004	↓ (MDD-Psy)

*The direction indicates the group that shows a positive (↑) or negative (↓) difference compared to the baseline.

### 3.3 Between-group activation differences during VFT

**Figure 2:**
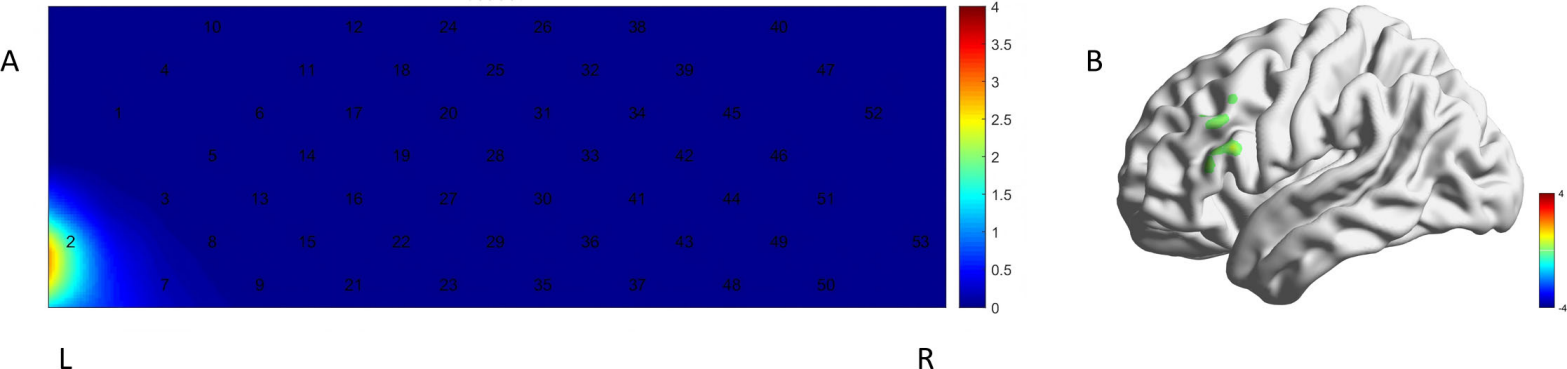
Frontal ROI Hemoglobin Changes During VFT **(A)** 2-D axial projection and **(B)** 3-D cortical rendering display the statistically significant contrasts between adolescents with MDD-NonPsy and those with MDD-Psy. Warm colors indicate regions where MDD-NonPsy > MDD-Psy, cool colors indicate regions where MDD-Psy < MDD-NonPsy.

**Figure 2** displays the FDR-corrected activation contrasts between adolescents with major depressive episode without psychotic symptoms (MDD-NonPsy) and those with psychotic symptoms (MDD-Psy). Two-dimensional axial and three-dimensional cortical renderings revealed a significant group difference at channel 2 located in the left dorsolateral prefrontal cortex (MNI: –60, 19, 18), where the MDD-Psy group showed a negative deviation from baseline (t = 2.780, pFDR = 0.006). The effect size for this difference was small-to-moderate (Cohen’s d = 0.34). It is important to note that this finding is restricted to a single channel and represents a modest effect; its robustness requires replication in independent samples.

### 3.4 Frontal ROI hemoglobin changes during VFT

Across the six frontal ROIs, HbR responses during the VFT remained close to baseline. After FDR correction, only the IFG_R exhibited a significant group difference (**Table 3**): the MDD-Psy group showed a negative shift (median –0.03 mM·mm, IQR -0.23-0.13) relative to the MDD-NonPsy group (0.00, IQR –0.12–0.15; z = -2.18, corrected p = 0.030). The absolute median difference between groups was 0.03 mM·mm, which is small in magnitude. Moreover, the interquartile ranges overlapped substantially, indicating considerable within group variability. Given the modest effect size and overlapping distributions, the physiological and clinical relevance of this HbR difference remains uncertain. No significant group differences were observed for any other ROI or for HbO responses (all p ≥ 0.166; see **Tables 3**, **4**). Median ΔHbO ranged from –0.01 to 0.03 mM·mm; none of the Mann–Whitney tests reached significance after correction (z = -1.01 to-0.05, p ≥ 0.311). The largest numerical divergence occurred in the left middle frontal gyrus (median 0.01 vs 0.03), yet the difference remained non-significant (z = -0.78, p = 0.438).

**Table 3:** De-oxyhemoglobin changes across frontal ROIs during the VFT task.

ROI	MDD-NonPsy (n = 110)	MDD-Psy (n = 163)	z	p
IFG_L	–0.02 (–0.19 – 0.08)	–0.01 (–0.21 – 0.15)	–0.10	0.917
MFG_L	–0.00 (–0.16 – 0.13)	0.00 (–0.11 – 0.10)	–0.81	0.417
SFG_L	–0.00 (–0.10 – 0.06)	–0.01 (–0.10 – 0.06)	–0.12	0.908
SFG_R	–0.01 (–0.12 – 0.07)	–0.01 (–0.10 – 0.07)	–1.39	0.166
MFG_R	–0.01 (–0.11 – 0.10)	–0.00 (–0.08 – 0.11)	–0.16	0.876
IFG_R	–0.00 (–0.12 – 0.15)	–0.03 (–0.23 – 0.13)	–2.18	0.030*

All variables deviated from normality (Kolmogorov–Smirnov, p < 0.001); values are reported as Median (IQR). Group differences were tested with the Mann–Whitney U test. *p < 0.05, **p < 0.01 (two-tailed).

**Table 4:** Oxygenated-hemoglobin activation across frontal ROIs during the VFT task.

ROI	MDD-NonPsy (n = 110)	MDD-Psy (n = 163)	z	p
IFG_L	0.00 (–0.11 – 0.13)	0.00 (–0.10 – 0.12)	–0.18	0.858
MFG_L	0.01 (–0.10 – 0.11)	0.03 (–0.04 – 0.11)	–0.78	0.438
SFG_L	0.01 (–0.05 – 0.12)	0.02 (–0.04 – 0.12)	–0.05	0.960
SFG_R	0.01 (–0.07 – 0.07)	0.01 (–0.08 – 0.11)	–0.73	0.468
MFG_R	–0.01 (–0.08 – 0.07)	0.00 (–0.08 – 0.07)	–0.36	0.716
IFG_R	0.02 (–0.10 – 0.12)	0.00 (–0.12 – 0.12)	–1.01	0.311

VFT, verbal fluency task; IFG, left inferior frontal Gyrus; MFG, middle frontal gyrus; SFG, superior frontal gyrus; IFG, inferior frontal gyrus; L, left hemisphere; R, right hemisphere.

### 3.5 Correlations of HbO at channel 2 and HbR in IFG_R with clinical severity

**Table 5** summarizes the Spearman correlations between hemodynamic responses at channel 2 (HbO) and the right inferior/medial frontal ROI (HbR) with clinical severity indices. Channel 2 HbO change did not correlate with any measure (ρ = -0.07 to 0.02, p ≥ 0.225). In contrast, higher de oxygenated hemoglobin in IFG_R was weakly but significantly associated with lower KDAS total scores (ρ = -0.14, p = 0.018) and lower restlessness sub scores (ρ = -0.16, p = 0.010); all other correlations remained non-significant (p ≥ 0.135). These correlation coefficients are small, indicating that the hemodynamic measure accounts for only approximately 2-3% of the variance in clinical scores. Thus, while statistically significant at the group level, the clinical relevance of these associations is limited.

**Table 5:** Spearman correlations between ch2/IFG_R(HBR) and clinical measures.

Clinical measure	ch2		IFG_R(HBR)	
	ρ	p	ρ	p
KDAS total score	–0.02	0.755	–0.14*	0.018
Lack of drive	–0.05	0.438	–0.08	0.196
Restlessness	0.02	0.768	–0.16*	0.010
BPRS total score	–0.06	0.331	–0.09	0.140
Anxiety–depression	–0.07	0.281	–0.05	0.417
Emotional withdrawal	0.01	0.897	0.04	0.498
Thought disturbance	–0.07	0.225	–0.08	0.169
Activation	–0.03	0.629	–0.09	0.135
Hostility	–0.01	0.914	–0.04	0.524

ρ, Spearman rank correlation coefficient; *p < 0.05, **p < 0.01 (two-tailed).

### 3.6 ROC-based discriminative performance of CH2 HbO and IFG_R HbR

**Figure 3:**
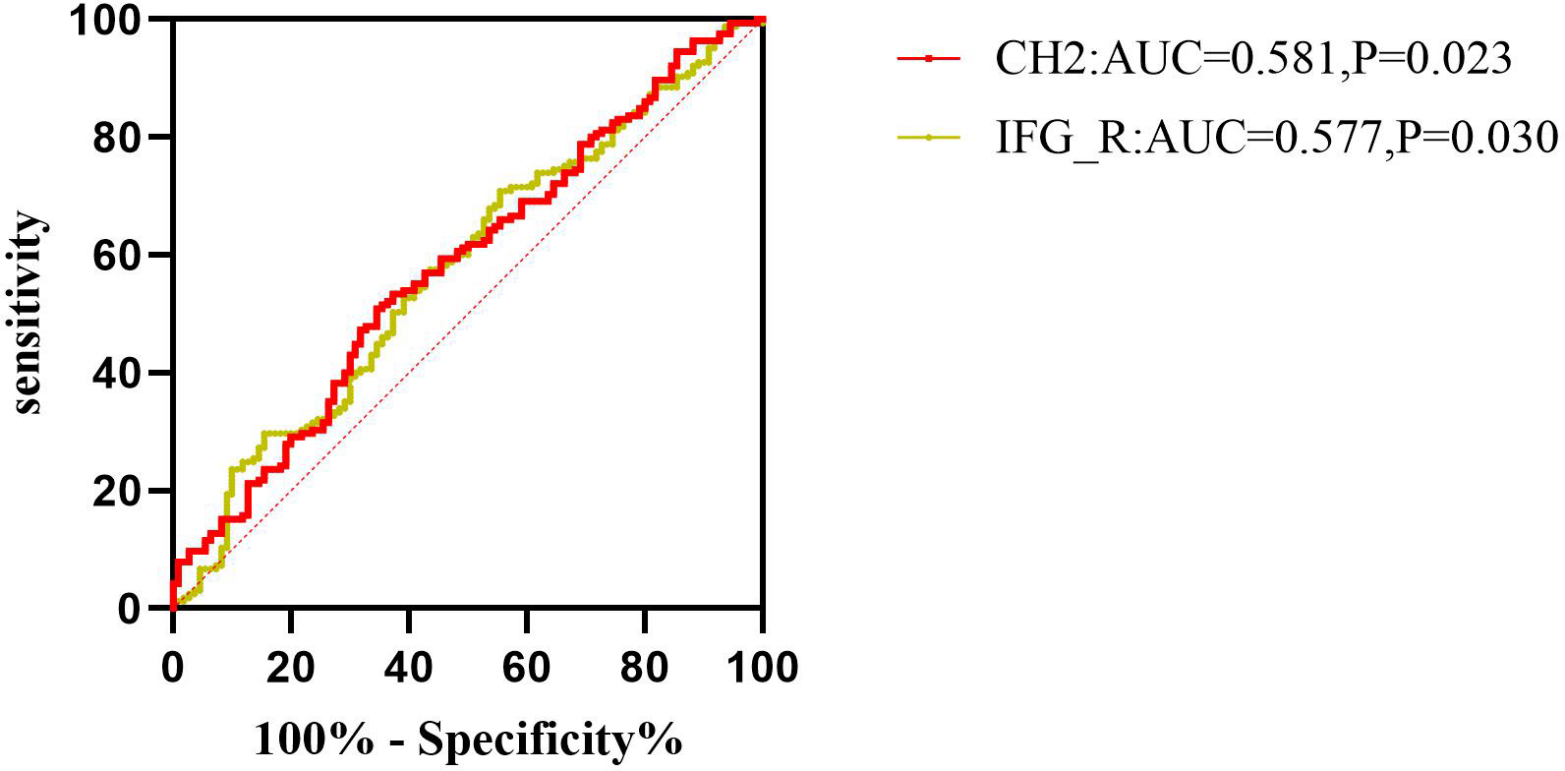
ROC Curves: CH2 HbO and IFG_R HbR for Psychotic-MDD Discrimination CH2 achieved an area under the curve (AUC) of 0.581 (P = 0.023), whereas IFG_R reached an AUC of 0.577 (P = 0.030).

**Figure 3** presents the receiver-operating characteristic curves for channel 2 oxygenated hemoglobin (CH2) and right inferior/medial frontal de-oxygenated hemoglobin (IFG_R) in classifying adolescents with psychotic versus non-psychotic major depressive episode. CH2 yielded an area under the curve (AUC) of 0.581 (95% CI: 0.512-0.650), with a statistically significant departure from chance (p = 0.023). IFG_R achieved a comparable AUC of 0.577 (95% CI: 0.508-0.646), also statistically significant (p = 0.030). Crucially, both AUC values are only marginally above chance level (0.50) and fall well below the threshold generally considered necessary for a clinically useful diagnostic test (typically ≥ 0.70–0.80). These results indicate that neither feature, alone or in combination, possesses sufficient discriminatory capacity to classify individual patients. The statistically significant p-values reflect the modest but detectable separation at the group level, but the substantial overlap in distributions precludes any claim of diagnostic utility. These findings should therefore be regarded as hypothesis-generating rather than evidence of a clinically applicable biomarker.

### 3.7 Exploratory sex-stratified analyses

Given the female-predominant sex distribution in our sample and potential sex differences in prefrontal hemodynamics, we conducted exploratory *post-hoc* analyses stratified by sex. The results are summarized in **Table 6**. Within the female subgroup (n=228), the patterns of reduced CH2 HbO activation and more negative IFG_R HbR in the MDD-Psy group remained directionally consistent with the primary full-sample analyses. In the male subgroup (n=47), differences for both biomarkers were in the same direction as in the full sample but did not reach statistical significance (p > 0.10), likely due to limited statistical power. These findings suggest that the identified neurovascular signatures are detectable in females but warrant further validation in larger, sex-balanced cohorts.

**Table 6:** Exploratory sex-stratified group comparisons of key fNIRS biomarkers.

Biomarker & subgroup	MDD-NonPsy (Median, IQR)	MDD-Psy (Median, IQR)	Mann-Whitney U/t-test statistic	p-value (uncorrected)
Channel 2 ΔHbO (mM·mm)				
Female (n=228)	0.005 (-0.050–0.062)	-0.012 (-0.071–0.043)	U = 5276, z = -1.98	0.048*
Male (n=47)	0.018 (-0.041–0.058)	-0.008 (-0.095–0.065)	U = 166, z = -0.72	0.471
IFG_R ΔHbR (mM·mm)				
Female (n=228)	0.002 (-0.115–0.152)	-0.032 (-0.225–0.128)	U = 5390, z = -2.22	0.026*
Male (n=47)	-0.005 (-0.158–0.121)	-0.025 (-0.295–0.105)	U = 154, z = -0.95	0.341

MDD-NonPsy, major depressive episode without psychotic symptoms; MDD-Psy, major depressive episode with psychotic symptoms; IQR, interquartile range; IFG_R, right inferior/medial frontal gyrus. * p < 0.05.

### 3.8 Sensitivity analyses for potential confounders

To assess whether the observed group differences in hemodynamic responses were confounded by demographic or clinical variables, we performed general linear models for channel 2 HbO and IFG_R HbR, including group (MDD−Psy vs. MDD−NonPsy) as the fixed factor and the following covariates: age, sex, medication status, illness duration (months), episode status (first/recurrent). After controlling for these variables, the main effect of group remained significant for both channel 2 HbO (F_1_,_260_ = 4.71, p = 0.031, η²_p_ = 0.018) and IFG_R HbR (F_1_,_260_ = 4.52, p = 0.034, η²_p_ = 0.017). These results indicate that the hemodynamic differences between MDD−Psy and MDD−NonPsy are not attributable to these potential confounders.

## 4 Discussion

In this exploratory study, we identified two fNIRS-derived neurovascular signatures that differ between, with modest effect sizes, adolescents with psychotic versus non-psychotic depression during verbal fluency: (i) a focal hypo-activation in the left dorsolateral prefrontal cortex (Channel 2; MNI –60, 19, 18) marked by reduced oxygenated hemoglobin (ΔHbO↓, pFDR = 0.004); and (ii) a right inferior frontal gyrus de-oxygenation deficit (ΔHbR↓) that is significantly more negative in MDD-Psy than MDD-NonPsy (median –0.03 vs. 0.00 mM·mm, pFDR = 0.030). Both features yield modest yet significant classification accuracy (left DLPFC HbO AUC = 0.581, right IFG HbR AUC = 0.577, p < 0.05), and the IFG signal correlates inversely with overall and restlessness severity (KDAS ρ = –0.14 to –0.16). Collectively, these preliminary findings suggest that reduced activation in the left DLPFC and diminished de−oxygenation in the right IFG may represent candidate neurophysiological signatures of psychotic depression in adolescents. However, given the modest effect sizes and the absence of external validation, these results should be considered hypothesis−generating rather than indicative of a ready−to−use biomarker. Importantly, because we did not include a healthy control group or other psychiatric comparison groups (e.g., schizophrenia, bipolar disorder), we cannot determine whether these signatures are specific to psychotic depression or represent a more general feature of psychosis or depression severity.

We caution that our fNIRS data cannot directly address neurotransmitter levels (e.g., NMDA, GABA) or large−scale network dynamics (e.g., DMN); the following interpretations are therefore speculative and offered only as hypotheses for future multimodal studies. Mechanistically, reduced left DLPFC activation in MDD-Psy is qualitatively consistent with prior evidence of altered glutamatergic metabolism in psychotic disorders (43–45) and with resting-state fMRI findings of altered DLPFC connectivity (46, 47), but our data do not permit causal inferences. The left DLPFC suppression we report may reflect a trans-diagnostic failure of top-down inhibition, but this remains to be tested using complementary modalities.

Meanwhile, MDD-Psy with decreased HbR in the right IFG aligns with previous reports of right IFG dysfunction in impulse control disorders (48) and non-suicidal self-injury (23), suggesting that right IFG abnormalities may be a trans-diagnostic correlate of inhibitory dysregulation across self-harm and psychosis spectra. Again, we emphasize that these links are hypothesis−generating and require validation with other neuroimaging techniques.

From a clinical perspective, the portability of fNIRS raises the possibility of future applications in risk stratification, but the present findings are far from translation. The portability of fNIRS enables rapid triage in emergency settings where MRI is unavailable. If replicated and validated in independent samples, the left-DLPFC hypo-activation at channel 2 could, in future studies, be explored as a neuronavigational anchor for individually titrated high-frequency rTMS (49), while the right-IFG HbR reduction identifies a complementary locus for inhibitory cTBS or cognitive-control training aimed at restlessness. At present, these remain speculative research directions. Importantly, these hemodynamic signatures classify MDD-Psy independently of the conventional KDAS/BPRS cut-offs, offering incremental prognostic value over symptom scales alone and reinforcing psychotic depression as a distinct endophenotype with divergent relapse trajectories (6).

Methodologically, the physiological dissociation between HbO and HbR complicates the interpretation of the fNIRS label. Our observation that right-IFG HbR decreases without concomitant HbO increases suggests vasodilatory uncoupling rather than a simple “activation” pattern. Simultaneous fNIRS-fMRI studies demonstrate that HbR is temporally aligned with the post-stimulus undershoot of the BOLD signal (50), corroborating the correspondence we noted with fMRI-BOLD (15, 39). The classification accuracy we observed (AUC ≈ 0.58) is modest and falls below the threshold generally considered necessary for a standalone diagnostic biomarker (≥ 0.70 (51)). Thus, our findings should be regarded as hypothesis-generating, and these hemodynamic measures are not yet suitable for clinical application. Future studies in larger, independent cohorts are required to determine whether combining fNIRS with clinical or other biological measures can improve predictive utility. Statistical power may be limited by unmeasured factors; Sensitivity analyses controlling for medication use confirmed the robustness of our findings; nevertheless, residual confounding by medication dose, duration, or specific agents cannot be entirely excluded. Additionally, sex−specific hemodynamic responses and SES could still modulate fNIRS signals (20). Finally, the 60-s verbal fluency task, while standard, lacks the ecological complexity of real-world executive demands; naturalistic paradigms such as story listening (52) may enhance ecological validity in future studies.

Our study compared only two subgroups of adolescents with major depressive disorder (MDD−Psy vs. MDD−NonPsy). We did not include a healthy control group, nor did we include other psychiatric comparison groups (e.g., schizophrenia, bipolar disorder with psychotic features, anxiety disorders). Consequently, we cannot determine whether the observed hemodynamic differences are specific to psychotic depression, generalizable to other psychotic disorders, or simply reflect differences in illness severity. Although our sensitivity analyses (Section 3.8) indicated that group differences remained significant after controlling for antipsychotic and antidepressant use, we cannot entirely exclude residual confounding due to medication dose, duration, or specific agent. Moreover, the cross−sectional design precludes causal inference regarding medication effects on hemodynamic responses.

Future studies should include multiple control groups—such as healthy adolescents and individuals with other psychiatric disorders (e.g., schizophrenia, bipolar disorder with psychotic features)—to establish the diagnostic specificity of the observed hemodynamic signatures. To dissociate illness−related from medication−related hemodynamic alterations, studies employing medication−naïve cohorts or longitudinal designs tracking patients before and after treatment initiation are essential. Such longitudinal approaches can also test whether these neurophysiological features predict treatment response, relapse, or clinical trajectory. Furthermore, prospective cohorts incorporating machine−learning−based neural prediction models, developed specifically for psychotic depression, may elucidate the prognostic value of these measures. Finally, multimodal integration represents a promising next step; for instance, synchronous fNIRS−EEG could capture the neuro−vascular oscillatory coupling that underlies the hemodynamic deviations observed in the present study (53).

## 5 Conclusions

Collectively, reduced left DLPFC activation and diminished right IFG de−oxygenation represent candidate neurovascular correlates that may help differ between psychotic from non−psychotic adolescent depression in this two-group comparison. Because we lacked healthy control and other psychiatric comparison groups, we cannot claim that these signatures specifically or diagnostically differentiate these conditions. While these preliminary signatures provide a bridge between descriptive psychiatry and mechanistic neuroscience, they are not yet ready for clinical translation. Replication in independent samples and prospective studies are essential before these measures can be considered for early triage or risk stratification. Future work should embed these biomarkers in longitudinal cohorts to track their evolution across illness stages and treatment epochs, integrate them with multimodal data streams—simultaneous EEG, inflammatory panels, and pharmacogenomics—to illuminate causal pathways, and embed them in adaptive clinical trials that use real-time hemodynamic feedback to guide personalized neuromodulation. Ultimately, translating these signatures into bedside decision algorithms will advance precision psychiatry and improve outcomes for the most vulnerable youth.

## Data Availability

The original contributions presented in the study are included in the article/**Supplementary Material**. Further inquiries can be directed to the corresponding authors.
