# Supplementary figures and images for: Reduced left dorsolateral prefrontal activation and right inferior frontal de-oxygenation differ between psychotic and non−psychotic adolescent depression during verbal fluency
Source: Front Psychiatry. 2026 Jun 2;17:1689631. doi: 10.3389/fpsyt.2026.1689631 (PMC13269331; doi:10.3389/fpsyt.2026.1689631)

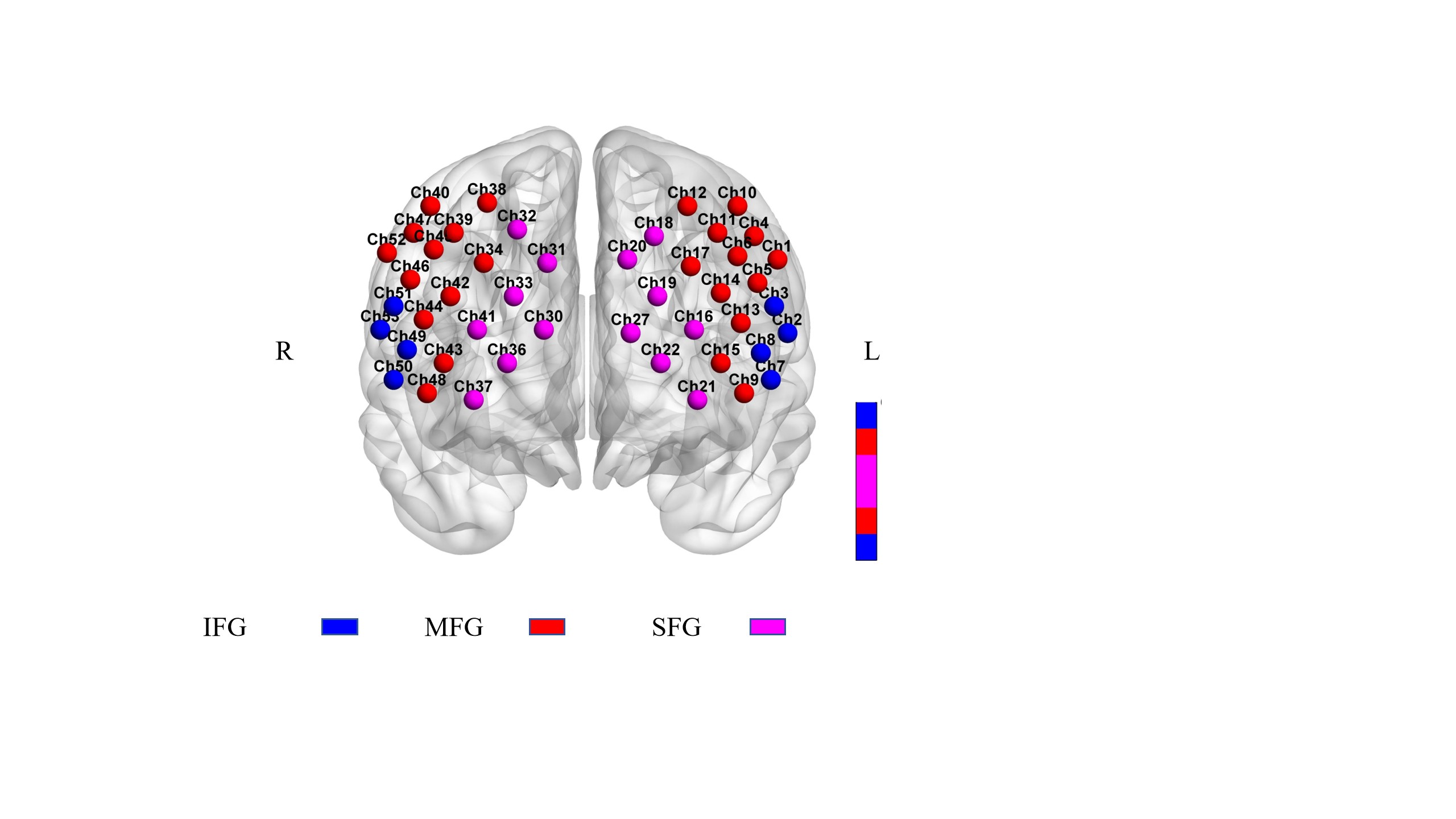

Supplement: Supplementary file 1 [file Image1.jpeg]
