# Supplementary material for: Reduced left dorsolateral prefrontal activation and right inferior frontal de-oxygenation differ between psychotic and non−psychotic adolescent depression during verbal fluency
Source: Front Psychiatry. 2026 Jun 2;17:1689631. doi: 10.3389/fpsyt.2026.1689631 (PMC13269331; doi:10.3389/fpsyt.2026.1689631)
Supplement: Supplementary file 2 [file Table1.doc]

**Table 1.1 MNI coordinates of fNIRS channels**

| **Channel Number** | **MNI coordinates** | | | **Broadmann area** | |
| --- | --- | --- | --- | --- | --- |
|  | **X** | **Y** | **Z** | **Name** | **Abbreviation** |
| Channel 1 | -57.3 | 6.3 | 40.3 | Left Pre-motor and supplementary motor area | PreM & SMA (L) |
| Channel 2 | -60 | 18.7 | 18.3 | Left Broca's area | Broca (L) |
| Channel 3 | -55.7 | 25 | 26 | Left Broca's area | Broca (L) |
| Channel 4 | -50.3 | 12.3 | 47.3 | Left Pre-motor and supplementary motor area | PreM & SMA (L) |
| Channel 5 | -50.7 | 29.7 | 33.3 | Left Broca's area | Broca (L) |
| Channel 6 | -44.7 | 31.7 | 40.7 | Left Dorsolateral prefrontal cortex | DLPFC (L) |
| Channel 7 | -55.3 | 35.7 | 4.3 | Left Broca's area | Broca (L) |
| Channel 8 | -52 | 40.7 | 12 | Left Broca's area | Broca (L) |
| Channel 9 | -47 | 52.3 | 0.3 | Left Frontal pole area | FPA (L) |
| Channel 10 | -45.3 | 11.7 | 55.7 | Left Pre-motor and supplementary motor area | PreM & SMA (L) |
| Channel 11 | -38.7 | 30.7 | 48.3 | Left Dorsolateral prefrontal cortex | DLPFC (L) |
| Channel 12 | -29.7 | 27.7 | 56 | Left Frontal eye field | FEF (L) |
| Channel 13 | -46.3 | 46.7 | 21.3 | Left Broca's area | Broca (L) |
| Channel 14 | -39.7 | 48.7 | 29.7 | Left Dorsolateral prefrontal cortex | DLPFC (L) |
| Channel 15 | -40.3 | 58.7 | 9.3 | Left Frontal pole area | FPA (L) |
| Channel 16 | -31.7 | 62 | 19 | Left Frontal pole area | FPA (L) |
| Channel 17 | -30.7 | 48.7 | 37.7 | Left Dorsolateral prefrontal cortex | DLPFC (L) |
| Channel 18 | -20.3 | 46.3 | 47.3 | Left Dorsolateral prefrontal cortex | DLPFC (L) |
| Channel 19 | -21.3 | 62.3 | 28.7 | Left Frontal pole area | FPA (L) |
| Channel 20 | -11.7 | 58.7 | 39.7 | Left Dorsolateral prefrontal cortex | DLPFC (L) |
| Channel 21 | -32.7 | 66 | -1.7 | Left Frontal pole area | FPA (L) |
| Channel 22 | -22 | 70.7 | 9 | Left Frontal pole area | FPA (L) |
| Channel 23 | -13 | 73 | -1.7 | Left Frontal pole area | FPA (L) |
| Channel 24 | -10.7 | 41.3 | 55.7 | Left Frontal eye field | FEF (L) |
| Channel 25 | 0.3 | 50.7 | 45.3 | Dorsolateral prefrontal cortex | DLPFC |
| Channel 26 | 11.7 | 41.7 | 56.3 | Right Frontal eye field | FEF (R) |
| Channel 27 | -13 | 70.7 | 18.3 | Left Frontal pole area | FPA (L) |
| Channel 28 | 0.7 | 63 | 27.7 | Frontal pole area | FPA |
| Channel 29 | 0.3 | 68 | 8.3 | Frontal pole area | FPA |
| Channel 30 | 13.3 | 71 | 19.3 | Right Frontal pole area | FPA (R) |
| Channel 31 | 12.3 | 59 | 39.3 | Right Dorsolateral prefrontal cortex | DLPFC (R) |
| Channel 32 | 21.3 | 47 | 48.7 | Right Dorsolateral prefrontal cortex | DLPFC (R) |
| Channel 33 | 22.3 | 63.3 | 29.3 | Right Frontal pole area | FPA (R) |
| Channel 34 | 31.3 | 49.3 | 39.3 | Right Dorsolateral prefrontal cortex | DLPFC (R) |
| Channel 35 | 13.3 | 73 | -1.7 | Right Frontal pole area | FPA (R) |
| Channel 36 | 23.7 | 71.7 | 8.7 | Right Frontal pole area | FPA (R) |
| Channel 37 | 34 | 67 | -2 | Right Frontal pole area | FPA (R) |
| Channel 38 | 29.7 | 28.7 | 57 | Right Frontal eye field | FEF (R) |
| Channel 39 | 40.3 | 31.3 | 48.3 | Right Dorsolateral prefrontal cortex | DLPFC (R) |
| Channel 40 | 47 | 12 | 56 | Right Pre-motor and supplementary motor area | PreM & SMA (L) |
| Channel 41 | 32.7 | 63.7 | 19.3 | Right Frontal pole area | FPA (R) |
| Channel 42 | 41.3 | 50.3 | 29.3 | Right Dorsolateral prefrontal cortex | DLPFC (R) |
| Channel 43 | 42.7 | 60.3 | 9.3 | Right Frontal pole area | FPA (R) |
| Channel 44 | 48.7 | 47.7 | 21.7 | Right Broca's area | Broca (R) |
| Channel 45 | 45.7 | 32.7 | 42.7 | Right Dorsolateral prefrontal cortex | DLPFC (R) |
| Channel 46 | 52.7 | 31.3 | 33.7 | Right Broca's area | Broca (R) |
| Channel 47 | 52 | 12.3 | 48.3 | Right Pre-motor and supplementary motor area | PreM & SMA (L) |
| Channel 48 | 48.3 | 54.7 | 0.3 | Right Frontal pole area | FPA (R) |
| Channel 49 | 54.3 | 41.7 | 12.7 | Right Broca's area | Broca (R) |
| Channel 50 | 58 | 35.7 | 4.3 | Right Broca's area | Broca (R) |
| Channel 51 | 57.7 | 26.7 | 26.3 | Right Broca's area | Broca (R) |
| Channel 52 | 59.7 | 6.3 | 41.7 | Right Pre-motor and supplementary motor area | PreM & SMA (L) |
| Channel 53 | 62 | 19.7 | 18.7 | Right Broca's area | Broca (R) |

MNI coordinates: the coordinates in Montreal Neurological Institute (MNI) space

**Table1.2 The channels correspond to the ROI partitions**

| ROI | channel |
| --- | --- |
| IFG-L | 2,3,7,8 |
| MFG-L | 1,4,5,6,9,10,11,12,13,14,15,17 |
| SFG-L | 16,18,19,20,21,22,27 |
| SFG-R | 30,31,32,33,36,37,41 |
| MFG-R | 34,38,39,40,42,43,44,45,46,47,48,52 |
| IFG-R | 49,50,51,53 |

ROI: region of interest
